# Online enquiries and health concerns – a survey of German general practitioners regarding experiences and strategies in patient care

**DOI:** 10.1007/s10389-023-01909-1

**Published:** 2023-04-12

**Authors:** Julian Wangler, Michael Jansky

**Affiliations:** Centre for General Medicine and Geriatrics, University Medical Center of the Johannes Gutenberg University Mainz, Am Pulverturm 13, 55131 Mainz, Germany

**Keywords:** Health anxieties, Doctor–patient relationship, Health information

## Abstract

**Aim:**

Increasingly at GP practices, patients appear who are extremely worried as a result of health information researched online and consequently affected by doubts and concerns. The study highlights GP attitudes and experiences with regard to this patient group. Moreover, it identifies strategies adopted by GPs to respond appropriately to worried or scared patients.

**Subject and methods:**

In the German federal states of Baden-Württemberg, Rhineland-Palatinate and Saarland, 2532 GPs were surveyed between June and August 2022. Owing to the explorative nature of the study, a descriptive analysis was conducted.

**Results:**

Of the total respondents, 77% deemed the current problem of internet-related health concerns to be a major challenge in everyday practice. The implications affect patients’ mental stability and expectations towards the doctor (esp. demand for further instrumental diagnosis, 83%). One doctor in five (20%) has experienced the termination of patient contact because the relationship with the patient was no longer possible due to the patient’s uncontrolled online information behaviour. To respond to worried or scared patients, the respondents generally ask certain patient groups about online research (39%) and take this into account in the doctor–patient discussion (23%). Furthermore, the respondents use a detailed explanation of the diagnosis and/or treatment (65%) and recommend websites that they consider reputable (66%). Some of the doctors prefer a joint examination of the information researched by the patient (55%) as well as to explain the benefits and risks of online research (43%).

**Conclusion:**

Many GPs demonstrate a high level of awareness and sensitivity with regard to extensive online research and potentially worried patients. It seems advisable to actively address the online search for information in the patient consultation to prevent possible negative effects on the doctor–patient relationship and to actively involve the patient. In this respect, it would also be worth considering expanding the medical history to include the dimension of online searching.

**Supplementary Information:**

The online version contains supplementary material available at 10.1007/s10389-023-01909-1.

## Introduction

Nowadays, researching health and disease-related topics on the internet is normal for many people (Lee et al. 2015; Dumitru et al. 2007). There is a chance that such online health research can improve patient education and thus increase understanding of a medical diagnosis and/or treatment (Powell et al. 2011; Link et al. 2022).

However, there is a risk of excessive internet research contributing to the emergence of entrenched health concerns, as it reinforces existing uncertainties or hypochondriac predispositions. It is therefore conceivable that discrepancies between medical (treatment) recommendations and online information could lead to heightened mistrust of doctors and to disorientation (Weaver et al. 2009). In particular, if symptoms, diagnoses or treatments are sought using search engines, there is a risk that users will find untrustworthy sites with erroneous information or will draw incorrect conclusions from what they have read (Lee et al. 2015).

The phenomenon of ‘cyberchondria’ is discussed as an extreme case of a negative effect from internet-based health information (McManus et al. 2014; Starcevic 2017). Based on the concept of hypochondria, it relates to a potentially extreme anxiety disorder or sensitivity with respect to one’s own state of health, caused in the long term by excessive internet research (Singh and Brown 2016). International studies indicate a link between intensity of online search behaviour and the use of medical appointments, diagnostic processes and health services (Eastin and Guinsler 2006; Muse et al. 2012; Murray et al. 2003; Te Poel et al. 2016).

It is a recurrent theme that patients are increasingly appearing at medical practices, who are extremely worried or scared as a result of health information researched online and consequently affected by doubts, worries and concerns (Baumgart 2010). So far, there is an extensive lack of empirical study findings here. A survey of around 800 doctors in various fields revealed that 78% frequently or occasionally found the effects of online self-information in everyday care counterproductive and detrimental (Bittner 2016). Around a third of doctors consider it right that patients who come to the practice with self-compiled information should be more involved in treatment decisions, whereas a further third advocate tyring to provide more detailed information for the same patients. Survey studies in the United States have shown that doctors facing pre-informed patients often feel restricted in their action options and no longer able to effectively make a difference to the patient (Murray et al. 2003; Xiang and Stanley 2017).

Because of their role as primary caregivers, GPs are especially affected by internet-related health concerns of patients. A qualitative study in 2020 provided evidence that GPs see the cyberchondria phenomenon as a growing challenge in everyday practice (Wangler and Jansky 2020). The problems raised include not only a considerable level of doubt and nervousness as well as hypersensitivity with respect to one’s own state of health, but at the same time also a low level of confidence in the doctor. In addition, compliance problems, the pronounced need for consultation/discussion on the part of the patient and the demand for further diagnosis are mentioned as difficulties. Attempts at stabilisation and further care are not always easy.

To date there is a lack of reliable studies that highlight the experiences of GPs with internet-related health concerns and how they respond to this patient group of sceptical or nervous patients. The present study accordingly pursued the following questions:

- How prevalent is extensive online research among GP patients? What effects does this have in the view/experience of the doctors?
- What experience do GPs have with patients who develop health anxieties as a result of preceding internet research in everyday practice? What do they find especially challenging?
- Which approaches do GPs chose in order to stabilise worried or scared patients or to prevent the occurrence of health concerns?

## Methods

In the summer of 2022, a comprehensive survey of GPs was conducted in three German federal states. This was designed as an online survey with a written letter by post.

### Investigation tools

On the one hand, the questionnaire was created on the basis of a literature search. This took into account the patient and doctor surveys mentioned. On the other hand, the results were fed into a qualitative preliminary study (Wangler and Jansky 2020), in the course of which 38 GPs in Rhineland-Palatinate were interviewed on the subject of cyberchondria. The results were decisive, in particular to firm up and concentrate the questionnaire, and they served specifically to generate two item sets (questions 7, 10). A pretest was conducted before use in the field.

The questionnaire consists of three content focuses: attitudes in respect of patients who conduct research online (questions 1 and 2, some of the item set in question 7); (behaviour) observations and characteristics of patients who conduct research online (questions 3 to 5, 8, 12); medical approaches to patients who conduct research online or who are worried or scared as a result of online research (questions 6, 10, 11, some of the item set in question 7).

### Recruitment

All 10,074 GPs who are active as practitioners in the German federal states of Baden-Württemberg (6664), Rhineland-Palatinate (2667) and Saarland (743) were invited in a written letter by post to take part in the anonymous survey between June and August 2022.

There were two main reasons for choosing these federal states. On the one hand, these federal states reflect the socio-demographic heterogeneity of all general practitioners in Germany, since urban and rural areas are equally represented. On the other hand, the selection of these federal states made sense for practical research reasons, since our institute regularly surveys general practitioners in these regions. We therefore have up-to-date contact lists, which helped to ensure a satisfactory response rate.

This was a one-off letter in which the doctors being surveyed received password-protected access to the online survey (no incentives).

### Sociodemographics and data analysis

Gender, age, practice environment, branch model and patients per quarter were collected as sociodemographic features.

After cleaning up the data set, the data were evaluated using SPSS 23.0 for Windows. Due to the explorative nature of the study, only a descriptive analysis was conducted.

## Results

Of the 2741 questionnaires not processed in full, 2532 forms completed in full went into the evaluation (response: 25%). The sample can be described as follows:

- Gender: 52% male, 48% female
- Practice environment: 49% medium-sized/large city, 51% rural/small-town
- Branch model: 46% individual practice, 48% group practice, 6% other
- Average age: 53 years

### Attitudes and experiences in respect of patients who conduct research online

Many of the respondents have a rather critical view of online research into health and disease-related topics; 53% assume that regular consultation of information on the internet tends to have negative consequences for the doctor–patient relationship (rather than positive consequences 19%, undecided 28%). Online research is regarded more favourably for clarification and health-related behaviour of patients (positive consequences 31%, negative consequences 41%, undecided 28%).

When asked about the specific effects on the doctor–patient relationship of patients increasingly obtaining health-related information from the internet, the majority of respondents observe that online research has considerable potential for confusion and uncertainty in their patients (cf. Table 1). This can lead to nervousness or to incorrect expectations on the part of the patient, owing to erroneous or contradictory statements, to a deterioration in compliance or even to an increasing willingness to self-medicate. Only a small proportion of respondents believe that regular searching on the internet could result in patients being better informed and having a deeper insight into medical diagnoses and procedures.

**Table 1:** The relationship between doctor and patient can change if patients increasingly or even regularly obtain information on health and disease topics from the internet. From your opinion or experience, which of the following points are correct? (Multiple selections were possible; N = 2.532)

Patients are confused and unsettled by information they get from the internet	84%
Patients become more nervous, more anxious	76%
Patients ask more questions	72%
Patients are more critical of the doctor	66%
Patients come to the office with the wrong expectations	61%
Patients check the doctor’s information, advice and diagnosis through internet research	49%
Internet research worsens patient compliance	45%
Patients tend to self-medicate	40%
Patients come to the office more often	38%
Patients are more willing to start conflicts with the doctor	36%
Patients have less trust in doctors	35%
Patients are better informed and can understand the doctor better	32%
Patients come to the doctor’s consultation hours in good time if they have complaints	17%
Patients avoid doctor visits more often	15%
Patients do not go to the doctor in time due to frequent internet searches	11%
Patients feel safer due to internet research	9%
Patients act more rationally due to regular internet research	7%

### Characteristics of patients who conduct research online

Of the GPs respondents, 69% estimate that 15% or more of their own patients frequently or have ever come to them with the results of their own internet searches (up to 10%: 25%). The researched information mainly concerns specific disease patterns (93%) or symptoms (88%), treatments (69%), diagnoses (57%) and new medications (45%).

In the experience of the respondents, the patients who undertake extensive research on the internet are mainly people under the age of 60 (84%); 45% name (previous) psychosomatic conditions as further attributes; 37% believe these are generally people with a higher level of education.

The respondents are familiar with the phenomenon that patients could be extremely worried or scared as a result of previous online research and consequently fear that they have a serious or even fatal illness, despite the fact that there are no corresponding indications from a medical perspective. As such, three quarters of all doctors state that they have already noticed such unfounded fear of serious illnesses based on acceptance of internet content either frequently (23%) or occasionally (51%) (rarely 24%). Moreover, 47% were of the opinion that such extreme forms of internet-related health concerns increased in the course of the COVID-19 pandemic either significantly (21%) or somewhat (26%). By their own admission, 20% have experienced patient care being terminated by the patient or by the GP because the patient’s behavior was so strongly affected by information from the internet that continued care was no longer possible.

As another item set confirms, 77% of respondents perceive patients who are extremely worried or scared as a result of internet research to be an increasing problem in everyday practice. Accompanying this, in the experience of 83% of doctors, is the fact that such patients tend to demand further instrumental diagnostics.

The concluding complex of the survey covers medical coping strategies and approaches to counter internet-related health concerns. Of the respondents, 39% say that they generally ask certain patient groups to what extent they have undertaken preliminary research on the internet before visiting the doctor; 23% state that they take this preliminary internet research into account very strongly or fairly strongly in the doctor–patient discussion. Furthermore, according to 46% of respondents, they frequently or occasionally recommend websites that they consider to be reputable and reliable sources of health-related information for patients.

GPs prefer certain approaches to help patients who are worried or scared as a result of internet research. As such, many doctors continue primarily to rely on a detailed explanation of the diagnosis and treatment; if necessary, they allow a longer consultation time (cf. Table 2). Most also consider it advisable to mention reputable health information on the internet or to hand out brochures with background information. By contrast, very few GPs consider it appropriate or practical to advise patients fundamentally against searching for information on the internet on their own initiative.

**Table 2:** In the case of patients whose overall psychological situation could be negatively influenced by internet research, the family physician can take certain measures to counteract the emergence of health anxieties. Which of the following points do you consider promising and practicable? (Multiple selections were possible; N = 2.532)

Giving the patient tips on reputable sources of information on the internet on relevant topics (e.g. certain health portals)	66%
Detailed explanation, e.g. on diagnosis and therapy, to prevent the patient from excessive or aimless internet research (if necessary, granting more consultation time)	65%
Joint discussion of the information or websites researched by the patient	55%
Handing out trustworthy information material (e.g. brochures)	54%
Extension of the typical anamnesis questionnaire to include the frequency of internet research on health and disease topics by the patient, so that the doctor can become aware of existing or emerging health anxieties at an early stage	45%
Basic discussion of the potential and risks of online research as part of the consultation	43%
Review of the information researched by the patient and consultation with the patient (e.g. for correction)	41%
In principle, advise patients not to search for information on the internet on their own initiative	18%

## Discussion

### Main findings and comparison with prior work

From the perspective of GPs, the survey confirms that doctors consider it to be normal that patients regularly obtain information about health and illness topics on the internet. However, three quarters of GPs observe as an increasing problem the fact that some of their patients acquire health concerns as a result of extensive online consultations. In the observation of the respondents, this trend increased in the course of the COVID-19 pandemic; this is reflected in the assumptions of relevant reviews (Wangler and Jansky 2020; Link et al. 2022; Starcevic et al. 2021).

The respondents’ experience of the effects of such independent health-related research is accordingly often negative in everyday practice, as they see primarily detrimental consequences for patients’ mental stability, expectations of the doctor or even willingness to self-medicate and compliance. One doctor in five now has personal experience of termination of one or more care relationships because the relationship with the patient was no longer possible due to the patient’s uncontrolled online information behaviour. However, it must be pointed out that these were assessments by the doctors. Nevertheless, such results are largely consistent with the qualitative preliminary study (Wangler and Jansky 2020) and with other studies in which doctors’ assessments have been sought concerning patients who undertake excessive research online (Murray et al. 2003; Baumgart 2010; Bittner 2016; Ahluwalia 2010). In the studies mentioned, there were also minorities of doctors who complained about considerable problems in doctor–patient communication, which in some cases led to a termination of medical care, be it on the part of the doctor or the patient.

At the same time, the results prove that many GPs have adapted to the phenomenon of cyberchondria and developed specific coping strategies. To respond to worried or scared patients or to prevent anxiety in the future, some doctors have started to take the precaution of asking certain patient groups about online research undertaken and to take this into account accordingly in the doctor–patient discussion. Overall, the medics surveyed rely on preventing internet-related health concerns primarily by taking enough time to explain the diagnosis and/or treatment and recommending specific websites that they consider to be reputable for follow-up or further research.

The behaviour of GPs is confirmed by relevant expertise on the doctor–patient relationship, which underlines the importance of the doctor having respect for the patient in the discussion (Kutscher and Seßler 2017; Mesko and Győrffy 2019). By taking into account the patient’s previous knowledge and by conveying the sense of actively contributing to the patient’s investigation, diagnosis or treatment, it is possible to incorporate additional, more comprehensive information and at the same to strengthen the medical practice. The consultation should follow the basic recommendations for dealing with patients with somatoform disorders (Tyrer 2018; Starcevic and Berle 2013). Ahluwalia et al. (2010) have previously highlighted the significance of psychosocial professionalisation of GPs for the successful treatment of worried or scared patients. Their qualitative study showed that GPs can learn to respond successfully to cyberchondria patients by using cognitive and behavioural techniques, allowing more consultation time and avoiding emotional responses to difficult patient groups.

If internet-related health concerns are too advanced and the GP is no longer able to stabilise such patients, it will be important to have an adequate range of low-threshold, psychosocial services to which doctors can easily and quickly make referrals. In view of the lack of available capacity in psychotherapy provision, compact online treatment could offer a valuable service, especially for patients affected by cyberchondria (Te Poel et al. 2016; Wangler and Jansky 2020; Tyrer 2018).

### Strengths and limitations

Owing to the limited number of cases and the regional recruitment focus, the study cannot claim to be representative. Moreover, it cannot be excluded that doctors with an interest in the subject will have participated in the survey to a greater extent. Nonetheless, on the basis of a satisfactory response rate, it was possible to obtain a heterogeneous sample, which extends across the breadth of GPs.

Finally it must be noted that the present survey is able to make no statements concerning how health concerns develop dynamically and are manifested individually among patients in connection with extensive internet research, i.e. to what extent they are fostered by certain conditions and interact with other (intervening) factors.

On the basis of clinical studies with patients who have health anxieties, several authors propose the development of a toolkit for GPs, so that those patients who have a tendency towards dysfunctional, pathological use of the internet can be filtered specifically and at an early stage and so that communication strategies can be developed (Eichenberg and Schott 2019; Tyrer et al. 2019).

## Conclusion

For doctors, the fact that patients obtain information about health and disease online before and after visiting the doctor is part of everyday care. This is not without consequences for the doctor–patient relationship; rather, it can have both a direct and an indirect impact on patients’ health behaviour, on their behaviour during the consultation and on compliance. In some cases, extensive internet research can trigger health concerns, which become entrenched in the long term. The medical handling of such ‘cyberchondria’ is certainly challenging. Many GPs are already aware of this. Overall, the findings indicate that GPs have begun to engage with the problem of internet-related health concerns and to look for solutions for (preventive) patient stabilisation.

In everyday practice, it seems sensible to respond actively to internet-based health research, to discuss its potentials and risks and to use it in the doctor–patient relationship. By responding to the patient’s research, the doctor is able not only to prevent potential anxieties but also to show respect. Both are beneficial for patient loyalty. In the light of this, it would also be worth considering expanding the medical history to include the dimension of (online) searching for information. Furthermore, consideration should be given to the fact that patients who have health concerns or are worried or scared as a result of contradictory information on the internet may require more consultation time. Not least, there should be some thought of strengthening the prominence of good, reputable information provision, not only for lay people but also among family practice specialists so that specific referrals to reliable health provisions can be made as directly and simply as possible (Bittner 2016).

## Supplementary information

ESM 1 (PDF 139 kb)

## Data Availability

All data generated or analysed during this study are included in this published article.

## Abbreviations

*GP(s)*: general practitioner(s)
