# Supplementary material for: Online enquiries and health concerns – a survey of German general practitioners regarding experiences and strategies in patient care
Source: Z Gesundh Wiss. 2023 Apr 12:1–7. Online ahead of print. doi: 10.1007/s10389-023-01909-1 (PMC10096108; doi:10.1007/s10389-023-01909-1)
Supplement: Supplementary file 1 — (PDF 139 kb) [file 10389_2023_1909_MOESM1_ESM.pdf]

1. The Internet offers patients many opportunities to find out more about health and illness topics on certain websites (e.g. health portals), to 'google' their own complaints or to exchange complaints with other users.

a) In your opinion, does this have more positive or more negative consequences for the doctor-patient relationship overall?

◯ Rather positive consequences ◯ Rather negative consequences ◯ Undecided

b) And how do you assess the consequences for the health behavior of patients?

◯ Rather positive consequences ◯ Rather negative consequences ◯ Undecided

2. The relationship between doctor and patient can change if patients increasingly or even regularly obtain information on health and disease topics from the Internet. From your opinion or experience, which of the following points are correct?

◯ Patients ask more questions
◯ Patients tend to self-medicate
◯ Patients come to the office more often
◯ Patients are better informed and can understand the doctor better
◯ Patients come to the office with the wrong expectations
◯ Internet research worsens patient compliance
◯ Patients feel safer due to internet research
◯ Patients become more nervous, more anxious
◯ Patients are more critical of the doctor
◯ Patients are confused and unsettled by information they get from the internet
◯ Patients avoid doctor visits more often
◯ Patients check the doctor's information, advice, and diagnosis through internet research
◯ Patients act more rationally due to regular internet research
◯ Patients are more willing to start conflicts with the doctor
◯ Patients come to the doctor's consultation hours in good time if they have complaints
◯ Patients do not go to the doctor in time due to frequent internet searches
◯ Patients have less trust in doctors
◯ Internet searches have no impact on patients

3. What percentage of your own patients approach you occasionally or frequently and ask you about health-related information they have researched themselves from the Internet?

◯ Less than 5% ◯ 5 to 10% ◯ 10 to 15% ◯ 15% to 20% ◯ More than 20%
◯ Nobody asks me about health-related information from the Internet (-> please go directly to **6.**)

4. About which topics did the respective patients research information on the Internet?

◯ Additional medical services ◯ Diagnostics ◯ Certain clinical pictures/diseases
◯ Disease symptoms ◯ Prevention ◯ Healthy lifestyle
◯ Health insurance services ◯ Care activities and care services ◯ Patient rights
◯ Therapies ◯ New drugs
◯ Other, namely: ____

5. What applies to most of these patients who inform themselves on the Internet?

◯ They are younger than 60 years ◯ They mostly live alone ◯ They have a higher level of education
◯ They are not employed ◯ They had a history of serious illness
◯ They have chronic illnesses ◯ These are patients with psychosomatic conditions
◯ Other, namely: ____

6. a) Do you ask specific patient groups whether preliminary research has been carried out on the Internet?

◯ Yes, usually ◯ Yes, now and then ◯ No (-> please go directly to 7.)

b) To what extent do you take this preliminary Internet research into account in your doctor-patient discussion?

◯ Very strongly ◯ Fairly strongly ◯ Less strongly ◯ Not at all

7. To what extent do you agree with the following statements?

	Totally agree	Rather agree	Rather disagree	Disagree at all
„Patients who do intensive research on health and illness topics on the Internet are usually better informed and more knowledgeable.“	◯	◯	◯	◯
„Patients who intensively use the Internet to find out more about their illness or symptoms tend to demand further instrumental diagnostics.“	◯	◯	◯	◯
„In everyday practice, family physicians do not have the time to deal with Internet information researched by patients or to comment on it.“	◯	◯	◯	◯
„Patients, greatly concerned or anxious by excessive Internet research, are an increasing problem in everyday practice.“	◯	◯	◯	◯
„Family physicians should signal more strongly that they consider themselves as point of contact for patients who seek information on health and illness on the Internet.“	◯	◯	◯	◯
„Physicians' fee schedules should reward physicians' activities related to providing advice on patients' online health research, as such advice is often time-consuming.“	◯	◯	◯	◯

8. Time and time again, there are reports of patients who are very insecure or frightened as a result of previous Internet research and, as a result, fear that they have a serious illness, although there is no corresponding information from a medical point of view. The term 'Cyberchondria' or 'Morbus Google' has become established for this.

How often have you noticed such an unfounded fear of serious illnesses, which is based on intensive Internet research, in your patients?

◯ Frequently ◯ Occasionally ◯ Rarely ◯ Never

9. From your own experience, to what extent would you say that, in the course of the COVID-19 pandemic, such Internet research-triggered health fears have increased among your patients?

Would you say…

◯ Significantly increased ◯ Somewhat increased ◯ Not increased
◯ Somewhat decreased ◯ Significantly decreased

10. In the case of patients whose overall psychological situation could be negatively influenced by excessive Internet research, the family physician may take certain strategies or measures to counteract the emergence of health anxieties.
Which of the following points do you consider promising and practicable?

◯ In principle, advise patients not to search for information on the Internet on their own initiative
◯ Detailed explanation, e.g. on diagnosis and therapy, to prevent the patient from excessive or aimless Internet research (if necessary, granting more consultation time)
◯ Giving the patient tips on reputable sources of information on the Internet on relevant topics (e.g. certain health portals)
◯ Handing out trustworthy information material (e.g. brochures)
◯ Joint discussion of the information or websites researched by the patient
◯ Review of the information researched by the patient and consultation with the patient (e.g. for correction)
◯ Extension of the typical anamnesis questionnaire to include the frequency of Internet research on health and disease topics by the patient, so that the doctor can become aware of existing or emerging health anxieties at an early stage
◯ Basic discussion of the potential and risks of online research as part of the consultation
◯ Other, namely: ____

11. How often do you recommend your patients certain websites to look up information about health and illness or even diagnoses and therapies?

◯ Frequently ◯ Occasionally ◯ Rarely ◯ Never

12. Do you have experienced patient care being terminated by the patient or by yourself, because the patient’s behavior was so strongly affected by information from the Internet that continued care was no longer possible?

◯ Yes ◯ No

13. There is a suggestion to expand the typical anamnesis questionnaire in such a way that the patient's frequency of Internet research on health and illness topics is systematically recorded. How useful do you think such a measure is?

◯ Very useful ◯ Rather useful ◯ Less useful or not useful at all

Finally, we need some statistical information from you. As with the rest of the questionnaire, all information is handled confidentially and anonymised.

Are you…

◯ Male ◯ Female ◯ Other

Your **age**: _____

Your **federal state**: ◯ Baden-Württemberg ◯ Rheinland-Pfalz ◯ Saarland

Your practice is located in a **municipality/town with…**

◯ More than 100,000 ◯ 20,000 to 100,000 ◯ 5,000 to 20,000 ◯ Fewer than 5,000 inhabitants

Which **practice model** best describes your practice?

◯ Single-partner practice (You are the only physician) ◯ Single-partner practice with contracted physicians

◯ Joint practice ◯ Medical care centre ◯ Other

How many patients does your practice treat every quarter?

◯ 500 to 750 ◯ 751 to 1,000 ◯ 1,001 to 1,500 ◯ 1,501 to 2,000 ◯ More than 2,000

Thank you for participating in this questionnaire!
